# piRNA-IPdb: a PIWI-bound piRNAs database to mining NGS sncRNA data and beyond

**DOI:** 10.1186/s12864-021-08071-6

**Published:** 2021-10-26

**Authors:** Odei Barreñada, Eduardo Larriba, Miguel A. Brieño-Enriquez, Jesús del Mazo

**Affiliations:** 1 Department of Cellular and Molecular Biology, Centro de Investigaciones Biológicas Margarita Salas (CIB-CSIC), Ramiro de Maeztu, 9, 28040 Madrid, Spain; 2 Institute of Bioengineering, University “Miguel Hernández”, 03202 Elche, Spain; 3 Department of Obstetrics, Gynecology and Reproductive Sciences, Magee-Womens Research Institute, University of Pittsburgh School of Medicine, Pittsburgh, PA USA

**Keywords:** piRNAs, PIWI, Immunoprecipitation, Database, microRNAs

## Abstract

**Background:**

PIWI-interacting RNAs (piRNAs) are an abundant single-stranded type of small non-coding RNAs (sncRNAs), which initially were discovered in gonadal cells, with a role as defenders of genomic integrity in the germline, acting against the transposable elements. With a regular size range of 21-35 nt, piRNAs are associated with a PIWI-clade of Argonaute family proteins. The most widely accepted mechanisms of biogenesis for piRNAs involve the transcription of longer precursors of RNAs to be processed, by complexes of proteins, to functional size, preferentially accommodating uridine residues at the 5’ end and 3’ methylation to increase the stability of these molecules. piRNAs have also been detected in somatic cells, with diverse potential functions, indicating their high plasticity and pleiotropic activity. Discovered about two decades ago, piRNAs are a large and versatile type of sncRNA and that remain insufficiently identified and analyzed, through next-generation sequencing (NGS), to evaluate their processing, functions, and biogenesis in different cell types and during development. piRNAs’ distinction from other sncRNAs has led to controversial results and interpretation difficulties when using existing databases because of the heterogeneity of the criteria used in making the distinction.

**Description:**

We present “piRNA-IPdb”, a database based uniquely on datasets obtaining after the defining characteristic of piRNAs: those small RNAs bound to PIWI proteins. We selected and analyzed sequences from piRBase that exclusively cover the binding to PIWI. We pooled a total of 18,821,815 sequences from RNA-seq after immunoprecipitation that included the binding to any of the mouse PIWI proteins (MILI, MIWI, or MIWI2).

**Conclusions:**

In summary, we present the characteristics and potential use of piRNA-IPdb database for the analysis of *bona fide* piRNAs.

## Background

piRNAs, the most recently discovered small non-coding RNAs, are essentially defined by their interaction with PIWI proteins. These molecules are single-stranded small non-coding RNAs with a regular size of about 21-35 nucleotides. They were first discovered in germ cells as defenders of genomic integrity in the germline, acting as post-transcriptional repressors of transposable elements [1]. Acting as guide RNAs for PIWI, piRNAs depend entirely in their bound with Argonaute-clade PIWI proteins to form a piRNA-induced silencing complexes. The processing of piRNAs starts from longer RNA molecules that are shortened during piRNA biogenesis, generating, preferentially, 5’ uridine residues and a 2’-O-methylated 3’-end, which increases the stability of these molecules [2]. A secondary piRNA biogenesis pathway has been proposed, in which a piRNA acts as template to recruit an antisense-complementary sequence to be processed into a new piRNA. Despite their being initially discovered and mostly studied in germ cells, piRNAs have also been detected in somatic cells [3] associated with diverse, not previously identified, potential functions, indicating their high plasticity and pleiotropic activity [2, 4–7], even though they are far from being fully characterized.

The distinction of piRNAs from other sncRNAs has led to controversial results and interpretation difficulties when existing databases are used because of heterogeneity in the criteria used to characterize piRNAs. Various databases are emerging to facilitate piRNA identification. But such bioinformatic tools could be considered too imprecise in regard to the basic consideration of what a piRNA is, in essence. Consequently, many sequences may be accumulated using different identifying criteria, such as are detected in NONCODE or RNAdb or others, such as piRNABank, piRNAQuest, IsopiRBank, or piRBase, that were initially considered piRNA specific. The problem has been the lack of a gold standard protocol for piRNA identification, so each database follows his own criteria and, in consequence, discrepancies appear among them. It seems, though, that in the claims made for the large number of sequences that populate these databases, a central point is being missed. By definition, piRNAs show an association with PIWI proteins [8]. Diverse characteristics have been associated with piRNA sequences, but the unique, invariable aspect of piRNAs should be their bond to PIWI proteins. In addition, several regions of other sncRNAs (tRNAs, rRNAs, snoRNAs, miRNAs) may share the essential features of piRNAs and act functionally as such [4, 9], but would not necessarily have to be considered as artifacts, as has been suggested [10], precluding potential functional mechanisms of regulation.

The aim of the present work is to elaborate and assess a database, piRNA-IPdb, based on the recent update of piRBase [11] and adjusting the piRNA identification criterion exclusively to *PIWI-bound* detected sequences after immunoprecipitation approaches in the mouse. Among the 21 species represented in the piRBase, the largest number of sets collected in the database corresponds to *Mus musculus*. Moreover, in vertebrates, mouse is the species with the highest number of piRNA datasets identified by immunoprecipitation with PIWI proteins. Overall, the present piRNA-immunoprecipitation database (piRNA-IPdb) can serve as a useful and trustworthy piRNA sequence resource for future piRNA research.

## Construction and content

### Database building and analysis

We have selected those sequences, from non-genetically modified mice at any developmental stage (from 10 days postpartum to adult testes or spermatids), exclusively obtained after immunoprecipitation with any PIWI protein (in mice, there are three PIWI proteins: MIWI, MILI, and MIWI2). From piRBase, we selected the datasets called: 5, 11, 12, 13, 31, 32, 33, 34, 35, 36, 37, 38, 60, 61, 72, 81, 82, 87, 88, 132, 133, 134, and 217 to extract piRNA ID, sequence, and reported read information (summarized in Table 1).

**Table 1:** The 23 datasets from mouse piRBase included for generation of the piRNA-IPdb

piRBase ID	Sequences	Total reads	Purification method	Tissue	PubMed ID	Accession	Series
5	3,482	No data	MILI IP	Testis	16,751,777	N/A	N/A
11	568,759	9,881,322	Miwi IP	Testes, C57BL/6 Adult Miwi +/+	22,121,019	GSM822760	GSE32184
12	720,903	8,723,133	Miwi IP	Testes, C57BL/6 P14 Miwi +/+	22,121,019	GSM822758	GSE32184
13	575,786	8,660,950	Miwi IP	Testes, C57BL/6 P20 Miwi +/+	22,121,019	GSM822759	GSE32184
31	1,068,849	7,028,316	Miwi CLIP	C57BL/6 adult testis	22,842,725	GSM684624	GSE27622
32	464,287	2,599,913	Miwi CLIP	C57BL/6 adult testis	22,842,725	GSM684625	GSE27622
33	118,015	625,561	Miwi CLIP	C57BL/6 adult testis	22,842,725	GSM684626	GSE27622
34	129,214	841,641	Miwi CLIP	C57BL/6 adult testis	22,842,725	GSM684627	GSE27622
35	249,590	4,978,836	Mili CLIP	C57BL/6 adult testis	22,842,725	GSM684620	GSE27622
36	187,442	5,177,881	Mili CLIP	C57BL/6 adult testis	22,842,725	GSM684621	GSE27622
37	167,997	3,670,855	Mili CLIP	C57BL/6 adult testis	22,842,725	GSM684622	GSE27622
38	194,293	3,487,124	Mili CLIP	C57BL/6 adult testis	22,842,725	GSM684623	GSE27622
60	334,652	471,657	Mili IP	16.5 dpc testis	18,922,463	GSM319956	GSE12757
61	1,086,617	1,940,312	Miwi2 IP	16.5 dpc testis	18,922,463	GSM319957	GSE12757
72	136,417	180,769	Mili IP	10 dpp testis	17,446,352	GSM179088	GSE7414
81	2,880,834	5,974,682	Mili IP	wild_type_1 E16.5 fetal testis	22,020,280	N/A	E-MTAB-730
82	3,229,065	7,409,965	Mili IP	wild_type_2 E16.5 fetal testis	22,020,280	N/A	E-MTAB-730
87	1,883,101	5,442,465	Miwi2 IP	wild_type_1 E16.5 fetal testis	22,020,280	N/A	E-MTAB-730
88	1,795,983	4,526,095	Miwi2 IP	wild_type_2 E16.5 fetal testis	22,020,280	N/A	E-MTAB-730
132	1,815,424	6,909,670	Miwi IP	adult testis	20,022,248	GSM475279	GSE19172
133	2,625,847	9,616,083	Mili IP	adult testis	20,022,248	GSM475280	GSE19172
134	879	No data	Miwi CLIP	elongating spermatids	24,787,618	N/A	N/A
217	3,517,319	17,503,453	MIWI CLIP	round spermatids	25,582,079	GSM1653802	GSE67683

From the whole piRBase data we filtered sequences for each of selected datasets (all from PIWI immunoprecipitation sequencing assays), with custom scripts (publicly available), obtaining sequences and expression data for each piRNA in each dataset. In parallel, the expression data was transformed from raw counts to count per million (dividing each sequence count by total count of the dataset and multiplying by one million) in order to normalize the diversity of sequencing methods of each dataset. With these data, we selected the most expressed 1 % of piRNA sequences and labeled them as “*highly expressed centile*” (“*hec”*).

The genome coordinates were obtained by aligning database sequences against the last published genome of the mouse (GRCm39/mm39) using Bowtie aligner, allowing one mismatch (-v1 parameter), and sorting the sequences with more than one possible valid alignment (-m1 option).

Scripts to generate the database are publicly available from project GitHub (https://github.com/OdBT/piRNA-IPdb_v2). Specific scripts were used for each of the analyses carried out, using mainly shell scripts for data analysis and R scripts [12] for data plotting.

We use the widely known NGS toolbox’s script (*basic-analyses.pl*) [13] to obtain the piRNA-IPdb sequence length distribution and the nucleotide composition. The detection of piRNA amplification cycle (ping-pong cycle) (reverse-complementary piRNA sequences overlapping 10 nucleotides) was performed using the *signature.py* Python script [14]. The presence of miRNAs related to piRNA-IPdb sequences was checked by mapping piRNA sequences against pre-miRNA, downloaded from miRBase using Bowtie sequence aligner and miRBase as microRNA database.

### Database details

For this piRNA-IPdb, a total of 18,821,815 unique sequences were pooled (obtained from 23 individual datasets from piRBase). Some interface functions have been integrated for the different piRNAs registered in the database with associated functions determined on the basis of the RepeatMasker database, including repetitive elements, different transposable elements (LINEs, SINEs, etc.), rRNAs, tRNAs, and, others (Table 1).

In the following sections, we will discuss the results of the exploratory analysis of this database, emphasizing the expected classical characteristics reported for piRNAs.

### Sequence length distribution

**Fig. 1:**
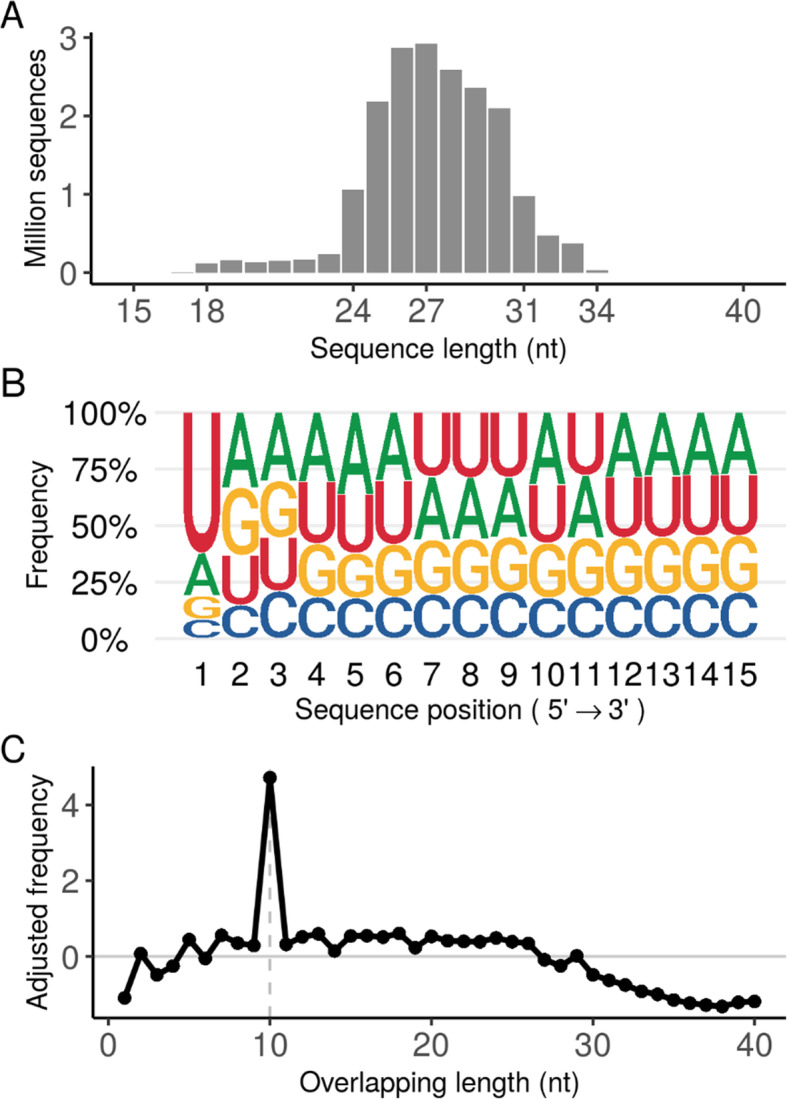
Database sequence analysis. **A**, Sequence length distribution of piRNA-IPdb. **B**, Nucleotide frequency of first 15 nucleotides from all sequences in the database. **C**, Frequency in the database of overlapping sequences among 10 nucleotides generated by potential *ping-pong* amplification cycles

The distribution of sequence lengths in this piRNA-IPdb showed that 90.23 % of sequences have lengths between 24 and 31 nt (Fig. 1A). These data are consistent with the sequences typically identified in the literature.

### Nucleotide composition

The pre-piRNA trimming process generates piRNAs that preferentially (but not unequivocally) start at 5’ with a uridine (1U-bias) [15]. We also checked the complete nucleotide composition of the first 15 nucleotides of all sequences. Results indicate that this 1U-bias is also present in piRNA-IPdb (Fig. 1B). Specifically, 62 % of the sequences start with this base.

### Ping-Pong cycle hallmark

A substantial number of piRNAs in the germline participate in a transposable element-mediated amplification process called a “*ping-pong*” pathway, initially characterized by the presence of piRNAs with the 1U-10A hallmark sequence [16], although this pattern is not unequivocally a consequence of the 1U-bias [17]. Using the *signature.py* Python script [14], we measured the quantity of antisense overlapping sequence pairs between database sequences. The results (Fig. 1C) confirmed that our piRNA database is also abundant in sequences showing this characteristic, with a high proportion of sequences overlapping in these 10 nucleotides.

### Measuring piRNA expression

**Fig. 2:**
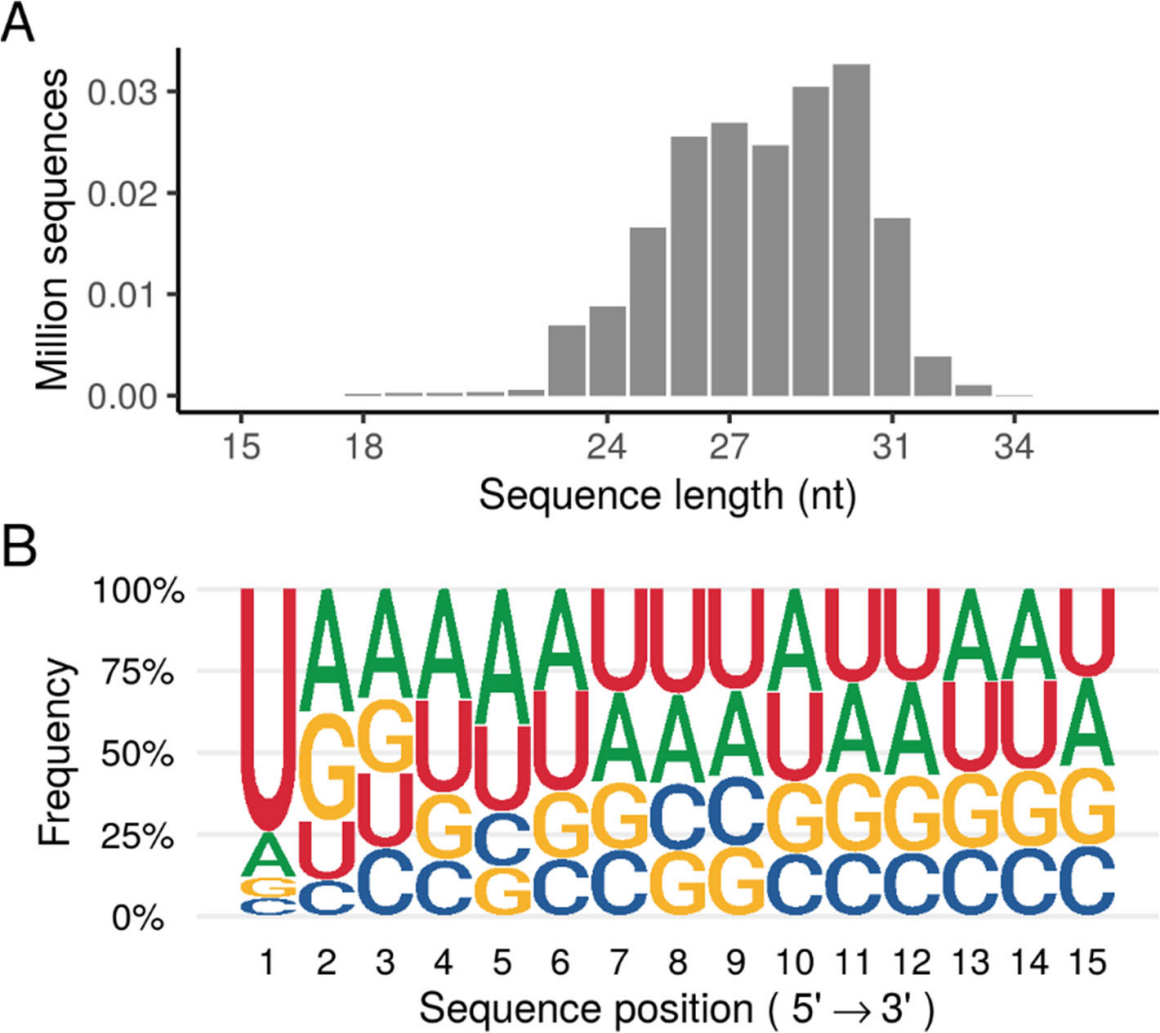
Sequence length distribution **A** and frequency of nucleotides of the first 15 nts in the sequences labelled as “*hec”* sequences **B**

We extracted information from the reads in each dataset (excluding ds5 and ds134, which did not contain such data). Due to the large variability in total library size for each dataset, reads were normalized using counts per million. The vast majority of piRNA sequences have a very low number of detected reads, although some groups of sequences displayed sequences with very well-detected expression. To highlight this feature, we labelled as FASTA files the most highly expressed 1 % of sequences with the “*hec*” label (as “*highly expressed centile”*). The sequence-length distribution of these sequences is shown in Fig. 2A. Compared to the whole database, *hec* sequence length distribution showed greater length and higher 1U-bias (Fig. 2B).

### Mapping piRNAs

The genome alignment of database piRNAs showed that 91.16 % of the sequences map, with only 1 mismatch allowance, to the mm39 genome database. However, 19.09 % of the total sequences map to multiple positions.

The sequence FASTA file is uploaded together with a BED file containing the genome coordinates of each piRNA with a unique aligned position.

### Web interface

In order to make the database accessible, we also created a web page available at (https://ipdb2.shinyapps.io/ipdb2/). The web page hosts the downloadable database files with a summary of the piRNA immunoprecipitation database.

## Utility and discussion

### Case study: Identifying miRNAs from the database sequences

As an exploratory example of interactive studies based on this database, we have evaluated the potential duality in the generation of miRNAs vs. piRNAs from an RNA precursor sequence with the capacity to generate either type of sncRNA, depending on their specific binding to AGO or PIWI proteins. Recent studies have raised doubts about the presence of other sncRNAs, such miRNAs, tRNAs, rRNAs, as contaminants in piRNA databases [10]. However, it is possible to identify cases in which both types of molecule (piRNA and other sncRNAs) could be bifunctional and even suggest alternative regulatory mechanisms to generate one or the other type. In fact, alternative, non-canonical pathways in the biogenesis of miRNAs have been reported [18–20]. Here, we evaluate that possibility for miRNAs/piRNAs from precursors that might generate such a situation.

**Fig. 3:**
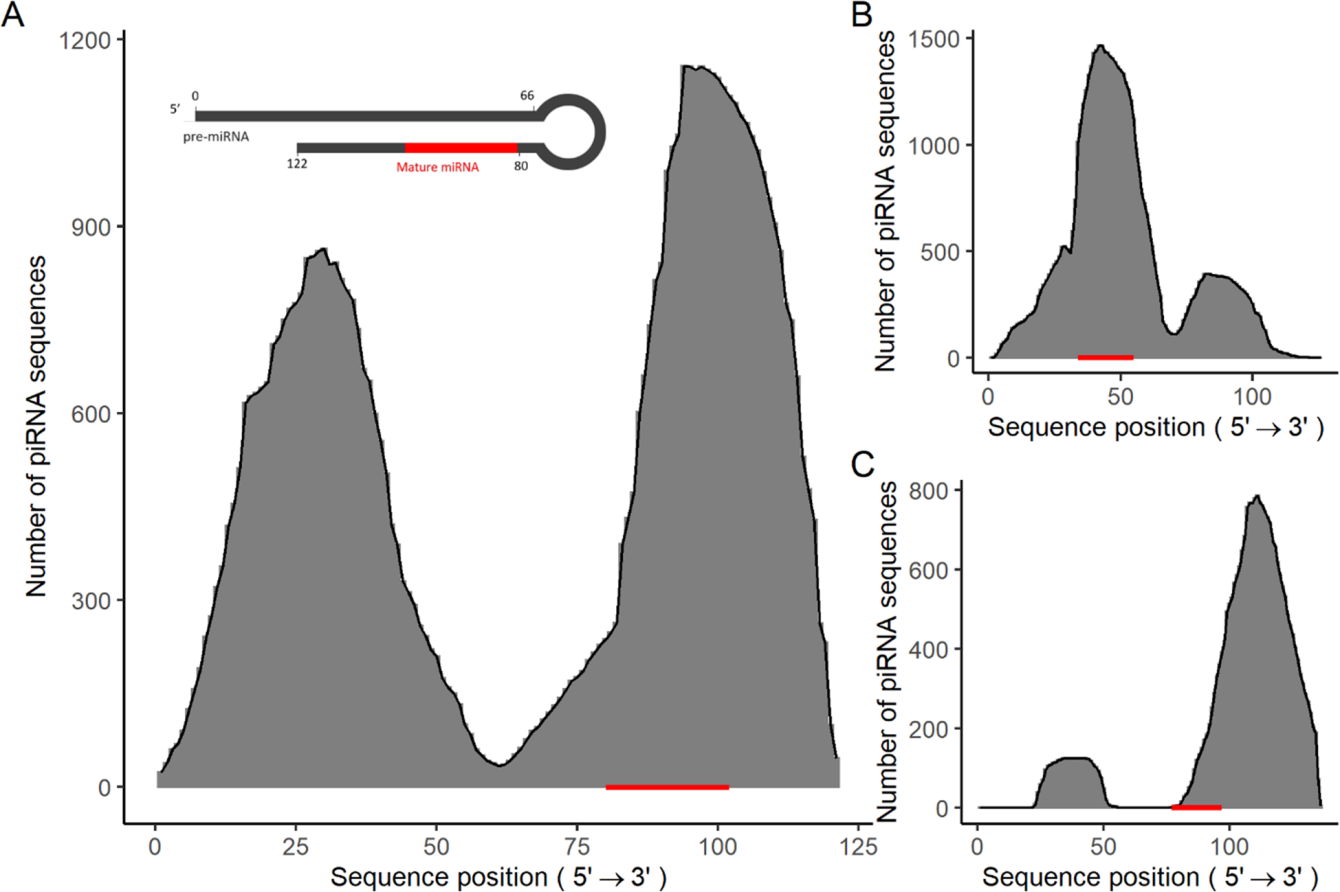
Examples of piRNA coverage against the top-mapping pre-miRNA sequences, figures show the number of different piRNAs sequences mapping pre-miRNA. Mature miRNA sequence positions are highlighted in red

Using miRBase as a reference, the sequences of the piRNA-IPdb have been mapped in the search for miRNAs. A total of 42,704 sequences (0.23 % of the total) potentially considered to be piRNA as well as miRNA have been detected, of which 607 showed identical sequences present in both databases, the rest being partially aligned or having just one nucleotide of difference (one mismatch). Some miRNAs have been aligned with up to 2000 different piRNAs (Table 2). A function has been integrated into the website database (https://ipdb2.shinyapps.io/ipdb2/, by which it is possible to directly recognize which is the coverage of piRNAs from a specific miRNA, simply by clicking on the function miRNA. Figure 3 shows some examples of coverage of piRNA sequences against miRNA sequences. This analysis, performed on the three miRNAs detected as most expressed, showed that the alignments of miRNA regions are coincident with the regions where the highest number of piRNA counts are detected over the pri-miRNA sequences (Table 3). This leads us to hypothesize that, since PIWI appears to be unable to bind to double-stranded RNAs, the generation of piRNAs should be previous to the stem-loop configuration of the pre-miRNAs—that is, a kind of by-product in piRNA biogenesis, where their precursors were in excess or the level of PIWI was limiting. Otherwise, regions corresponding to the loop or single-stranded ends of pri-miRNAs would correspond to higher abundances of piRNAs after the processing of such pri-miRNAs.

**Table 2:** microRNAs with higher numbers of piRNA sequences mapping against each of them

Number of piRNAs	miRNA
2253	mmu-mir-1194
2251	mmu-mir-3470b
1010	mmu-mir-3473f
877	mmu-mir-468
864	mmu-mir-3473 h
823	mmu-mir-5098
821	mmu-mir-3471-2
724	mmu-mir-6240
544	mmu-mir-3470a
447	mmu-mir-7222

**Table 3:** Summary of total reads by dataset with percentage of mapped miRNAs. Note that some of the datasets report a high percentage of miRNAs. No specific miRNA is significantly shared among the datasets

Dataset	Reads	Unique Seq	miRNA mapping percentage
5	No data	3,482	0.06
11	3,878,745	568,759	0.08
12	8,067,670	720,903	0.14
13	8,423,133	575,786	0.10
31	4,604,886	1,068,849	0.08
32	1,920,319	464,287	0.09
33	472,914	118,015	0.08
34	669,819	129,214	0.11
35	2,273,190	249,590	0.18
36	3,237,581	187,442	0.16
37	1,695,287	167,997	0.25
38	2,239,101	194,293	0.20
60	177,504	334,652	1.05
61	891,594	1,086,617	0.27
72	84,533	136,417	0.79
81	4,433,943	2,880,834	0.09
82	5,723,639	3,229,065	0.08
87	4,408,653	1,883,101	0.17
88	3,633,216	1,795,983	0.23
132	4,639,135	1,815,424	1.59
133	7,374,889	2,625,847	0.65
134	No data	879	0.11
217	6,729,883	3,517,319	0.07

## Conclusions

In this study we present the creation of the piRNA database “piRNA-IPdb”, a database with 18,821,815 sequences that are detected after immunoprecipitation with PIWI proteins, we check that these piRNAs meets the regular characteristics of *bona fide* piRNAs, and we demonstrate the utility of such database for piRNA analysis at the case study.

## Data Availability

The datasets generated and analyzed during the current study are available as GitHub repository, at https://ipdb2.shinyapps.io/ipdb2/ webpage.

## Abbreviations

piRNAs: PIWI-interacting RNAs
sncRNAs: Single-stranded small non-coding RNAs
NGS: Next-generation sequencing
piRNA-IPdb: piRNA-immunoprecipitation database
*hec*: Highly expressed centile
miRNAs: MicroRNAs

## References

[1] Aravin AA, Naumova NM, Tulin AV, Vagin VV, Rozovsky YM, Gvozdev VA (2001). Double-stranded RNA-mediated silencing of genomic tandem repeats and transposable elements in the D. melanogaster germline. Curr Biol.

[2] Ozata DM, Gainetdinov I, Zoch A, O’Carroll D, Zamore PD (2019). PIWI-interacting RNAs: small RNAs with big functions. Nat Rev Genet.

[3] Ross RJ, Weiner MM, Lin H (2014). PIWI proteins and PIWI-interacting RNAs in the soma. Nature.

[4] Barreñada O, Fernandez-Perez D, Larriba E, Brieño-Enriquez M, del Mazo J (2020). Diversification of piRNAs expressed in PGCs and somatic cells during embryonic gonadal development. RNA Biol.

[5] Liu Y, Dou M, Song X, Dong Y, Liu S, Liu H (2019). The emerging role of the piRNA/piwi complex in cancer. Mol Cancer.

[6] Huang X, Wong G (2021). An old weapon with a new function: PIWI-interacting RNAs in neurodegenerative diseases. Transl Neurodegener.

[7] Tamtaji OR, Behnam M, Pourattar MA, Hamblin MR, Mahjoubin-Tehran M, Mirzaei H (2020). PIWI-interacting RNAs and PIWI proteins in glioma: molecular pathogenesis and role as biomarkers. Cell Commun Signal.

[8] Girard A, Sachidanandam R, Hannon GJ, Carmell MA (2006). A germline-specific class of small RNAs binds mammalian Piwi proteins. Nature.

[9] Larriba E, del Mazo J (2018). An integrative piRNA analysis of mouse gametes and zygotes reveals new potential origins and gene regulatory roles. Sci Rep.

[10] Tosar JP, Rovira C, Cayota A (2018). Non-coding RNA fragments account for the majority of annotated piRNAs expressed in somatic non-gonadal tissues. Commun Biol.

[11] Wang J, Zhang P, Lu Y, Li Y, Zheng Y, Kan Y (2019). piRBase: a comprehensive database of piRNA sequences. Nucleic Acids Res.

[12] R Core Team (2020). R: A language and environment for statistical computing.

[13] Rosenkranz D, Han CT, Roovers EF, Zischler H, Ketting RF (2015). Piwi proteins and piRNAs in mammalian oocytes and early embryos: From sample to sequence. Genom Data.

[14] Antoniewski C (2014). Computing siRNA and piRNA overlap signatures. Methods Mol Biol.

[15] Stein CB, Genzor P, Mitra S, Elchert AR, Ipsaro JJ, Benner L (2019). Decoding the 5’ nucleotide bias of PIWI-interacting RNAs. Nat Commun.

[16] Gunawardane LS, Saito K, Nishida KM, Miyoshi K, Kawamura Y, Nagami T (2007). A slicer-mediated mechanism for repeat-associated siRNA 5’ end formation in Drosophila. Science.

[17] Wang W, Yoshikawa M, Han BW, Izumi N, Tomari Y, Weng Z (2014). The initial uridine of primary piRNAs does not create the tenth adenine that Is the hallmark of secondary piRNAs. Mol Cell.

[18] Xie M, Steitz JA (2014). Versatile microRNA biogenesis in animals and their viruses. RNA Biol.

[19] Lemus-Diaz N, Ferreira RR, Bohnsack KE, Gruber J, Bohnsack MT (2020). The human box C/D snoRNA U3 is a miRNA source and miR-U3 regulates expression of sortin nexin 27. Nucleic Acids Res.

[20] Stribling D, Lei Y, Guardia CM, Li L, Fields CJ, Nowialis P (2021). A non-canonical microRNA derived from the snaR-A non-coding RNA targets a metastasis inhibitor. RNA.

